# Oral malignant melanoma: a rare case with unusual clinical presentation

**DOI:** 10.11604/pamj.2015.22.113.7773

**Published:** 2015-10-09

**Authors:** Elneel Ahmed Mohamed Ali, Musadak Ali Karrar, Abeer Abdalla El-Siddig, Azza Zulfu

**Affiliations:** 1 Oral & Maxillofacial Surgery, Faculty of Dentistry, University of Khartoum, Sudan; 2 OMF Surgery, National University, Sudan; 3 Faculty of Medicine, Pathology Department, Taibah University, Medina, KSA; 4 Department of Pathology, Khartoum Teaching Hospital, Ministry of Health, Khartoum, Sudan

**Keywords:** Sudan, oral malignant melanoma, epithelial neoplasm, unusual presentation

## Abstract

Primary Oral malignant melanoma is a rare tumor with an indigent prognosis. This is a case report of 47-year-old Sudanese female diagnosed as Oral malignant melanoma of the mandible with an unusual pattern of growth and clinical presentation. Furthermore, a possibility of intraosseous origin is suggested.

## Introduction

Malignant melanoma is an epithelial lesion originated from malignant transformation of melanocytes or melanocytes precursor that present in the mucosa [1]. It was first described by Weber in 1859 and later was named as “melanotic sarcoma” by Lucke in 1869 as quoted by Pandey [2]. In Sudan, the malignant melanoma represents 0.7% of all oral neoplasm [3]. It is a very aggressive tumor with high tendency to early metastasis regionally to lymph nodes and distance place where lung and liver being the most common sites [4]. The purpose of this case report is to describe unusual clinical features of malignant melanoma.

## Patient and observation

**Figure 1:**
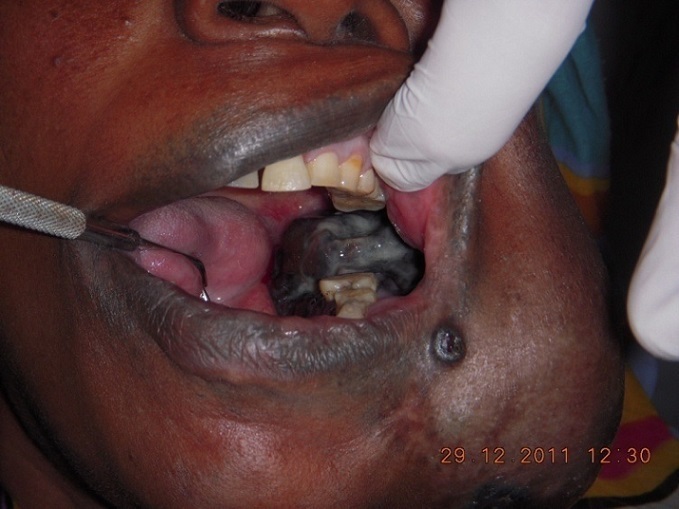
Clinical picture showing an intraoral black discolored lesion with extraoral expansion. Note the black nodule in the left commissar

A 47 years old female referred to the department of Oral and Maxillofacial Surgery from a rural hospital complaining of painful swelling at left side of the mandible of two years duration. Started as painless small swelling which increased gradually in size with history of tooth exfoliation. The patient reported recent numbness in the lower lip and she was not aware of any intraoral pigmentation prior to this complain. The past medical history was insignificant and there is no family history for such lesion. On clinical examination, there was facial asymmetry with pigmented nodule at left angle of the mouth and the over lining skin was tethered. Intraorally, there was huge tender black discolored swelling extended bucco-lingulay as well as anterio-posteriorly from area distal to 36 to the pterygomandibular trigone with palpable submental and bilateral submandibular lymph nodes (Figure 1). A very careful clinical examination was done to the patient skin to exclude any other pigmented lesion and no remarkable signs were detected.

**Figure 2:**
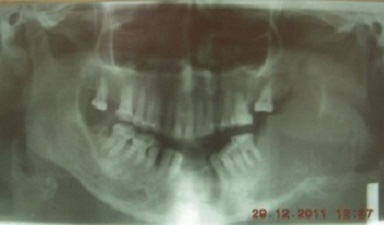
Orthopantomogram showing ill defined radiolucency associated with pathological fracture

**Figure 3:**
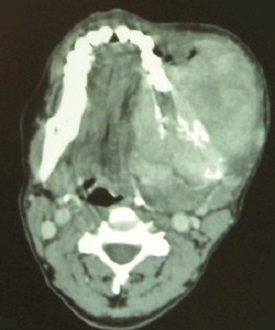
Axial CT scan showing destruction of the bone and soft tissues enhancement that extended buccally and lingually

**Figure 4:**
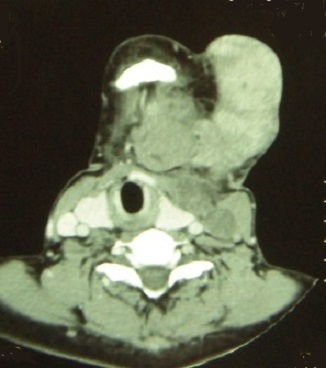
An axial CT scan view showing tethered skin and left jugulodigastric lymph node enlargement with central necrosis

**Figure 5:**
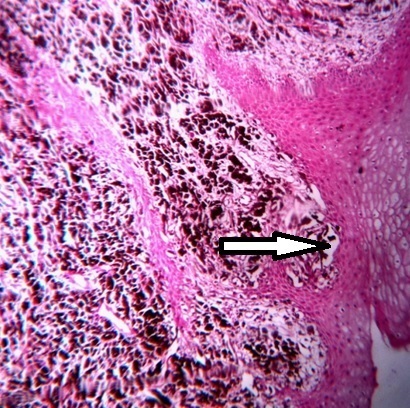
Showing an infiltrative tumor spreading through the submucosa with junctional activity (arrow). The tumor cells shows spindling and melanin production

**Figure 6:**
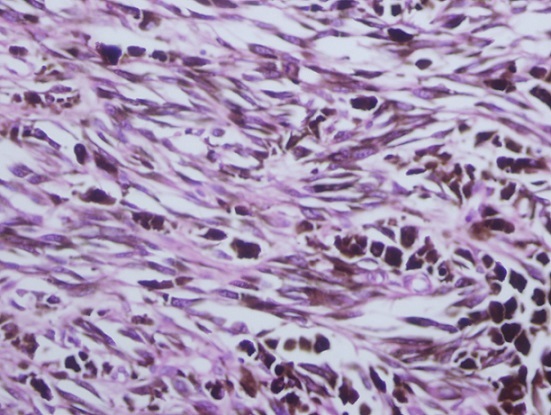
Showing a tumor consisting of melanin producing spindle and pleomorhic cells are having large nuclei

Orthopantomography showed an ill defined radiolucency in the left mandibular angle associated with pathological fracture (Figure 2). Axial CT image showed a cortical bone destruction, soft tissues expansion and necrotic left jugulodigastric lymph node (Figure 3 and Figure 4). The histological examination of incisional biopsy revealed an infiltrative tumor spreading through the submucosa with evidence of junctional activity. The tumor consisting of melanin producing spindle and pleomorphic cells having large nuclei (Figure 5 and Figure 6). A diagnosis of oral malignant melanoma was established. Chest x-ray and abdominal ultrasound were done to exclude any distance metastasis and no abnormalities were found. After insuring that the patient and her family understand her medical condition, the treatment options and the prognosis, the decision was made to refer the patient to receive chemotherapy and radiotherapy. Unfortunately, two months later the patient passed away.

## Discussion

Primary malignant melanoma of the oral cavity is rare lesion which accounts for 0.7% of all neoplasms of oral cavity in Sudan [3]. The metastatic lesion to oral cavity is even more rare with tongue being the most common site [5]. The risk factors that have been proposed are Cigarette smoking, denture irritation, alcohol consumption and immune suppression all of which are excluded in this case [6]. Furthermore, it has a racial predilection with higher incidence in Japanese and black people. Most of these occur in male with 3:1 ratio. It most commonly affects adults and rarely seen in children with peak incidence at sixth decade of life. The most common involved sites are mucosa of the hard palate followed by maxillary gingivae. In contrast to this case, where a female with lesion at the angle of the mandible in the fourth decade of life is presented, which make it a very rare case. It is may be asymptomatic in the early stages but in most cases it is presented with late stage and accompanied with nodular enlargement, pain, bleeding or ulceration [2]. The two most common clinical growth patterns are lentiginous and nodular and they may appear at the same lesion. Moreover, in the late presentation, the nodular type become prevalent and the lentiginous part disappear [7]. In this case, there is only vertical growth and the OPG view showed that the tumor favor the bone instead of spreading to the soft tissues. The authors suggest that either the tumor have different pattern of growth from that described in the literature or has different site of origin like intraosseous ectopic melanocytes.

Recently, in 2010, the American joint committee on cancer adopted a staging system for head and neck mucosal melanoma. This staging system reflects the aggressiveness of this tumor entity. There is no stage 1 or 2 and started from stage 3 as mucosal disease and stage 4 for moderate and advance diseases [8].

The diagnosis of mucosal malignant melanoma of the oral cavity can be done on H&E stained sections like skin melanoma. Malignant melanoma in H&E sections shows cells of different shapes, spindle cells, rounded and pleomorphic cells may be seen. The cells may show high degree of cellular atypia with abundant eosinophilic cytoplasm, large nuclei and prominent eosinophilic nucleoli. Mitotic activity might be prominent with atypical forms. Mucosal malignant melanoma usually shows junctional activity and upward migration of the malignant cells. The mucosal malignant melanoma of the oral cavity is classified as follows: Insitu oral mucosal melanoma, invasive oral mucosal melanoma and mixed insitu and invasive lesions. There is a group of border line lesions termed atypical melanotic proliferations [8].

The treatment of choice is surgical excision with surgical margins. According to Zitelli et al, the safety margins should be at least 1.5cm for the lesion in head and neck melanoma or a margin of 2.5 cm for melanomas larger than 3 cm in diameter [9]. The hidden location and rich vascularization, of oral mucosa make its malignant melanoma usually presents at advanced stage with locoregional and/or distance metastasis with 75% metastasis to lymph nodes and 50% to distance locations with liver and lung being the most common sites [10].

Spontaneous regression of cutanous malignant melanoma has been reported [11] but it is very rare and it was excluded in this case because of presence of junctional activity in histopathological picture (Figure 6). Greene et al proposed three criteria in the diagnosis of primary oral melanoma: the presence of malignant melanoma in the oral mucosa, exclusion of melanoma in any other primary site and histopathological determination of junction activity, all of which were evident in this case [12].

## Conclusion

Intraoral mucosal malignant melanoma is a very rare carcinoma. It has an aggressive course of growth with poor prognosis. The pattern of growth favors mainly the soft tissues. To the author's knowledge, no case of intraosseous malignant melanoma has been reported before. Here, the possibility of such origin is suggested.
